# The cost of space independence in P300-BCI spellers

**DOI:** 10.1186/1743-0003-10-82

**Published:** 2013-07-29

**Authors:** Srivas Chennu, Abdulmajeed Alsufyani, Marco Filetti, Adrian M Owen, Howard Bowman

**Affiliations:** 1 Department of Clinical Neurosciences, University of Cambridge, Cambridge, UK; 2 Centre for Cognitive Neuroscience and Cognitive Systems, University of Kent, Canterbury, United Kingdom; 3 Department of Computer Science, Taif University, Taif, Saudi Arabia; 4 Brain and Mind Institute, University of Western Ontario, London, Canada

**Keywords:** RSVP Speller BCI, Matrix P300 Speller BCI, Space-independence, Gaze-independence, Rapid serial visual presentation

## Abstract

**Background:**

Though non-invasive EEG-based Brain Computer Interfaces (BCI) have been researched extensively over the last two decades, most designs require control of spatial attention and/or gaze on the part of the user.

**Methods:**

In healthy adults, we compared the offline performance of a space-independent P300-based BCI for spelling words using Rapid Serial Visual Presentation (RSVP), to the well-known space-dependent Matrix P300 speller.

**Results:**

EEG classifiability with the RSVP speller was as good as with the Matrix speller. While the Matrix speller’s performance was significantly reliant on early, gaze-dependent Visual Evoked Potentials (VEPs), the RSVP speller depended only on the space-independent P300b. However, there was a cost to true spatial independence: the RSVP speller was less efficient in terms of spelling speed.

**Conclusions:**

The advantage of space independence in the RSVP speller was concomitant with a marked reduction in spelling efficiency. Nevertheless, with key improvements to the RSVP design, truly space-independent BCIs could approach efficiencies on par with the Matrix speller. With sufficiently high letter spelling rates fused with predictive language modelling, they would be viable for potential applications with patients unable to direct overt visual gaze or covert attentional focus.

## Background

There are now a number of relatively mature methods for interfacing the brain with modern computer systems and devices by interpreting electrical brain activity in real-time, most commonly using non-invasive electroencephalography (EEG). In particular, EEG-based Brain Computer Interfaces (BCIs) have been explored extensively over the last two decades, based on detectable changes observed at the scalp in response to motor imagery Event-Related Desynchronisation (ERD) [1-3], Steady State Visual Evoked Potentials (SSVEPs) [4], Slow Cortical Potentials (SCPs) [5-7] and the P300 Event Related Potential (ERP) [8]. These techniques variously assume motor, neural and cognitive capacities of the user. For example, SCP-based BCIs rely on feedback that teaches users to modulate their own brain rhythms to produce slow (low-frequency) EEG changes that can be detected in real-time. Users of typical SSVEP-based approaches, on the other hand, need to shift their gaze to one amongst many spatially separate flickering patches, and select by holding gaze upon one such patch. Users of the well-studied P300-based letter matrix BCI select letters in a 2-D grid by fixating on them and counting flashes [9]. This raises the key issue of spatial dependence in BCI designs; that is, what cognitive and residual motor capacities does use of a particular BCI method require [10,11]? Most importantly, the extent of a method’s dependence on such capacities governs its domain of applicability, since the degree of a user’s disability will rule out certain approaches. For example, a patient without control of gaze (for example, patients in a completely locked-in state) will not be able to use an SSVEP system employing spatially offset patches.

Accordingly, there has been much recent interest in BCIs that are completely independent of eye gaze and more specifically, whether such independent BCIs can achieve bit rates that make them feasible. However, there are different levels at which independence can be considered. This is because, even if patients are unable to shift *overt* visual attention (i.e. eye gaze), they might, even with a fixed gaze, be able to spatially shift their spotlight of attention within the visual field, through so called *covert* attention. Indeed, a number of psychophysics experiments on visual attention rely upon this capacity, e.g. attentional capture [12] or the Posner task [13]. In addition, selective brain damage to candidate visual attention areas, such as the Superior Colliculus [14], Pulvinar Nucleus of the thalamus [15] or the Temporo-Parietal Junction [16] could result in a variety of hybrid deficits crossing the spectrum of covert and overt visual attention, e.g. Neglect patients exhibit intact vision, but typically impaired attention deployment specifically to the left visual field [17]. Toward applications with such patient groups, researchers have recently investigated BCI designs that are gaze-independent. These designs rely on the user’s ability to shift covert (rather than overt) attention in visual space, and detect the presence of consequent P300 ERPs [18-22], motion VEPs [23,24] or changes in alpha band power [25].

However, there may exist patients with deficits that manifest as an inability to spatially shift and hold *either* overt or covert attention, but spare vision at fixation. In addition, even if holding covert attention at a non-foveal location might be possible for some patients, it is not clear to what extent this would induce visual fatigue detrimental to usability. Thus, it is interesting to consider BCIs that go beyond gaze independence, and are completely independent of spatial shifts in attention. That is, could a practical BCI be developed in which all stimuli are presented exactly at foveal fixation?

An SSVEP-based method that would seem indeed to be fully space-independent is the SSVEP interface proposed by Allison [26], which presents overlaid horizontal and vertical gratings flickering at distinct frequencies. The user then endeavours to perceptually foreground the desired grating, generating a corresponding SSVEP signature and providing a binary communication channel. The approach though, only realised one bit per minute or less in communication throughput (significantly less than the space-dependent alternative it is compared with in Allison [26]). This then raises the question of whether a wholly space-independent BCI could be devised with a bit rate above one per minute, and also of how that rate would compare to those of existing space dependent BCIs. In other words, what is the cost of requiring space independence? These are the questions we explore here.

More specifically, we will consider a particular method for realising a space-independent BCI, viz. presenting all stimuli at fixation (with each stimulus rapidly replacing its predecessor) in, so called, Rapid Serial Visual Presentation (RSVP), and detecting user selections via the P300 ERP. Users “search” an RSVP stream such that the vast majority of non-salient items remain sub-threshold, while most of the salient items “breakthrough” into conscious awareness. It is this breakthrough that we detect as the P300. Empirical investigations have demonstrated that this search can be based on both intrinsic salience, e.g. a threatening word when searching for job words [27], and (explicit) volitionally-prescribed task set [28,29]. The latter capability is exploited in the RSVP BCI. For example, at a particular moment, the BCI user might be searching a stream of letters for a “K”, which becomes the task set [30]. Demonstrated that ALS patients could use a simple space-independent BCI with 4 serially presented choices by generating P300s. More recently, BCI designs have exploited this idea to demonstrate the viability of fully-fledged RSVP spellers [31,32] to perform online classification of P300s generated by RSVP [33]. Extending from this work [34], successfully tested an online RSVP BCI coupled with predictive language modelling with a Locked-in Syndrome (LIS) patient. The ‘Center Speller’ proposed by [19] further optimises the design of space-independent spellers, by employing a two-level procedure to first select a letter group presented in a circle around fixation, and then select a letter within that group.

These developments bode well for practical applications of space-independent spellers. However, in choosing a BCI design for a particular patient, it is worth considering the trade-offs inherent in opting for true spatial independence (see [35] for a comprehensive review of BCIs from this perspective). Toward informing this choice, our objective in this article is to comparatively assess the RSVP and Matrix spellers in an offline setting. These two designs effectively lie at either end of a potential spectrum of space-independence within which gaze-independent BCIs represent intermediate levels. In particular, we are interested in how key differences in the target frequency and stimulus layout in these spellers feed into the time course of consequent EEG dynamics and classifiable information therein. To make a fair and general sable comparison, we employ ‘plain vanilla’ , standard instantiations of the spellers, while keeping all other experimental parameters the same. We will show that the RSVP design performs considerably better than the SSVEP-based overlaid gratings design [26], and has an accuracy on par with the Matrix speller [9]. Further, we will demonstrate that in sacrificing space, the RSVP approach in its basic form has lower throughput, but at the same time is less dependent on space-dependent ERPs for its performance. In doing so, we provide a current assessment of the cost of space-independence in P300-based BCI spellers.

## Methods

### Participants

The study was approved by the ethics committee of the Faculty of Sciences at the University of Kent. It included eleven participants (five female, six male), all of whom were students at the University of Kent and ranged in age from 19–26. All participants were right-handed, free from neurological disorders, and had normal or corrected-to-normal vision. They provided written consent and were paid for their participation.

### Stimulus presentation

Participants were asked to spell words by counting occurrences of the constituent letters. Alphabet stimuli were presented on a 20” LCD screen with a refresh rate of 60 Hz and a resolution of 1280 × 1024, placed at a distance of 60 cm from the participant. Stimuli were presented in uppercase white colour on dark grey background, and subtended approximately 2.5 degrees of visual angle.

### Stimuli

Participants were presented six 5-letter English words (‘RIGHT’ , ‘WORLD’ , ‘BLACK’ , ‘QUEST’ , ‘FLAME’ and ‘HEAVY’) in two modes of presentation, RSVP and Matrix, making up a total of twelve words they were asked to spell. The words were chosen so as to have equal lengths, and to ensure that all letters of the alphabet were proportionally represented. The order and mode in which these words were presented was randomised to prevent any unintended performance difference between the two modes. Before presenting any of the words, participants undertook a practice session, in which the word ‘HI’ was presented once in each mode. Data from the practice session were excluded from any analysis.

### RSVP mode

Each word comprised five *blocks* (one per letter), with successive letters being designated as target in each block. Within each block, there were a number of stimulus *repetitions*, varying randomly between 8 and 12. This randomisation ensured that there was a behavioural counting task required of participants (see below). Each repetition consisted of an RSVP stream of 25 uppercase English letters flashed in random order and without repetition at the centre of visual fixation (the letter X was excluded because it was already used as a fixation cross before the beginning of the stream). The target letter was presented exactly once in each repetition. The Stimulus Onset Asynchrony (SOA) for each letter was 166 ms, with an on time of 133ms and off time of 33 ms.

### Matrix mode

The overall structure of the Matrix mode was the same as RSVP, with blocks of letters making up a word. The main change was that instead of presenting letters in RSVP, we employed the well-known 2-D speller matrix originally proposed by Farwell and Donchin [9]. Here, participants were shown a 5 × 5 matrix of the same 25 letters as in RSVP. A repetition was defined as the successive flashing of all 5 rows in random order, followed by all 5 columns in random order. As with RSVP, each flash lasted 133ms, and was followed by an off time of 33 ms. This resulted in a key duration difference between the RSVP and Matrix modes: while a single repetition in RSVP consisted of 25 letter presentations lasting 4.15 s (= 25 × 166 ms), an equivalent repetition in Matrix lasted 1.66s (= 10 × 166 ms).

### Experimental task

In both presentation modes, there were no pauses between repetitions, but there were pauses after each letter block. At the beginning of each word, participants were asked to count the number of times they saw each target letter being presented or flashed (depending on the mode). Since participants were told that they would be asked to report the number of targets they counted, the randomisation of the number of repetitions in a block allowed us to behaviourally measure whether they attended equally well in both modes.

**Figure 1:**
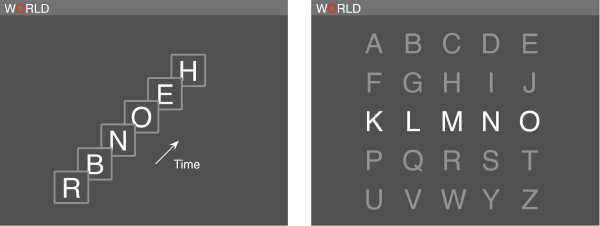
**Example of stimulus presentation in RSVP (left) and Matrix (right).** In both modes, 2nd letter ‘O’ (the target letter) of the word ‘WORLD’ is being spelt, and is highlighted in red. In RSVP mode, letters were presented in rapid succession at central fixation. Participants counted occurrences of the target in the sequence. In Matrix mode, rows and then columns of the letter display were rapidly flashed on and off. Participants counted the number of times the target was flashed.

In RSVP mode, participants were asked to focus their gaze and attend to the entire RSVP stream, while in Matrix mode they were instructed to focus their gaze and attention only on the target letter located within the matrix. These instructions were followed by the current word being displayed at the top of the screen. After 2 s, the target letter to be counted was highlighted in red. Alongside, either a fixation cross (‘X’; RSVP mode) or the spelling matrix (in light grey colour; Matrix mode) was presented. 5 s following this, presentation of letters began, either in RSVP or by flashing rows and columns of the matrix (see Figure 1). At the end of each block, participants were presented with a list of numbers between 8 and 12 in random order, followed by a ‘None of Above’ option. They were instructed to use only the up, down and enter keys on a standard UK keyboard to select an option in an unspeeded fashion. Once they had done so, the next letter in the current word was highlighted as the target letter and the next block began. Participants were requested to avoid eye blinks or any body movements during a letter block. They were permitted to blink and relax at the end of each block.

### EEG setup collection

Electroencephalographic data was recorded from 7 scalp electrodes (F_z_ , C_z_ , P_z_ , P_3_, P_4_, O_1_ , O_2_) within the standard 10–20 system and the 2 earlobes (A_1_ and A_2_) using a Brain Products QuickAmp recorder (Brain Products, Munich, Germany). The 7 scalp electrodes were chosen based on a previous study [36], which showed that a similar montage (F_z_ , C_z_ , P_z_ , O_z_ , PO_7_ , PO_8_) produced the best P300 b classification performance. We chose P_3_, P_4_, O_1_ and O_2_ instead of PO_7_, PO_8_ and O_z_ as we were additionally interested in recording bilateral occipital steady-state responses to RSVP and Matrix mode stimuli. The left mastoid was set to be the ground electrode. The sampling rate was 1000 Hz, and the data were average referenced and bandpass filtered online during recording, between 0.3-85 Hz. Electrooculograms (EOG) were recorded from the left and right eyes using two bipolar horizontal and vertical EOG electrodes. Impedances were always below 7 kOhm (2.27 kOhm on average).

### Pre-processing

Continuous EEG data from each participant was first down sampled to 250 Hz and then low-pass filtered at 40 Hz. Individual epochs were then extracted by segmenting the data between -200 ms and 800 ms relative to the time of each letter presentation. Linear trends were removed from each epoch and they were adjusted to have an average of zero baseline activity between −200 and 0 ms. Approximately 7500 epochs were generated in RSVP mode (25 letters × ~10 repetitions × 5 letters × 6 words) including 300 target epochs. Similarly, there were around 3000 epochs (10 flashes × ~10 repetitions × 5 letters × 6 words) in Matrix mode, including 600 target epochs. Artefactual epochs containing peak-to-peak variation greater than 100 μV in EOG or EEG channels were excluded from epoch-level classification analysis. The epochs were finally re-referenced to the linked mastoid electrodes, which, along with the EOG electrodes, were then removed from the data.

In order to make a fair comparison between the two modes, we re-combined epochs in the Matrix mode, so as to make each epoch therein equivalent to an epoch in RSVP mode. Specifically, we took the 10 epochs in a Matrix mode repetition and combined each one of the 5 epochs corresponding to a row flash with each of the 5 corresponding to a column flash, by averaging every such pair. Each of these 25 new Matrix mode ‘pair-average’ epochs thus generated was the same length as RSVP mode epochs. Furthermore, of these 25, only the one averaging over the two epochs corresponding to the target row and column flash was marked as the new target epoch, while the remaining 24 were marked as non-target epochs. Importantly, this pair-averaging ensured that, in either mode, a roughly equal number of target and non-target epochs were available for classification analyses, and that performance estimates could be validly compared. Furthermore, each of these new pair-average epochs could be considered to be ‘informationally equivalent’ to their RSVP mode counterparts, as data from two flashes in each repetition (one row and one column) are required to uniquely detect the selection of a letter in Matrix mode.

To generate features for the classification analyses, data was first downsampled to 25 Hz. Then the 20 samples between 0-800 ms (or 300-600 ms in follow-up analysis) from the 7 scalp channels in each epoch were concatenated to form one ‘observation’ of the feature set, consisting of 140 features. Finally, feature vectors were converted to normalized Z-scores by subtracting out the feature-wise means and then dividing by the respective standard deviations.

### Epoch-level classification

Stepwise linear discriminant analysis (SWLDA; Draper and Smith [37]) and Receiver Operating Characteristic (ROC) analysis was employed to estimate the optimal discriminability of targets from non-targets based on the single-trial P300 evoked in the two presentation modes. SWLDA has been shown to work well in EEG classification, providing an effective trade-off between complexity and speed [38,39].

In order to assess the classifier’s generalisability, we used 10-fold cross validation to calculate accuracy. Specifically, during each fold, a different 10% of target and 10% of non-target epochs were excluded for testing. Then a SWLDA classification algorithm with a feature entrance tolerance of 0.1 and exit tolerance of 0.15 [38] was trained on the remaining target and non-target epochs. The algorithm returned a coefficient weight and p-value for each feature, indicating its efficacy as a predictor. These weights were sorted by their p-values, and the 60 (or fewer) best features, i.e.,with the lowest p-values,which were also included in the regression model generated by SWLDA, were then selected. The weights of these best features were then used to calculate classification estimates of the same training epochs. ROC analysis of these estimates was used to calculate ROC curves and the optimal signal detection threshold (or ‘criterion’) that maximised the difference between the number of true and false positives. This key step improved overall classification accuracies by correcting for the classifier’s detection bias, due to the significant difference in the number of epochs of each class included for training. Next, the 60 classifier weights were used to calculate classification estimates of the previously excluded test epochs. The threshold was then applied to these estimates to decide classification outcomes of the test epochs, and accuracy for the fold. This entire procedure was repeated 10 times, by excluding a different 10% of epochs each time. Overall cross-validated accuracy, threshold and areas under the ROC curves were estimated as averages of the values calculated in each fold.

### Letter-level classification

To simulate and compare performance of the two modes in an online BCI setting, we calculated the average number of letters correctly identified in each presentation mode using a 50:50 train-test procedure. All epochs, including artefactual ones excluded above, were considered for this analysis, to generate a realistic estimate of online performance. For each participant, a SWLDA classifier was trained on epochs from the first three words in each mode, and tested with epochs from the last three words. For each letter block in the tested words, classification estimates for each instance of the 25 letters presented/flashed were separately averaged across the first 8 repetitions making up the block (as all letter blocks had at least 8 repetitions). The letter that got the highest average estimate in a block was considered to be the most likely target letter, and marked as identified correctly if it matched the actual letter in the word the participant had been asked to spell. With this procedure, we estimated the letter detection accuracy and ITR in each presentation mode. Information Transfer Rate (ITR) or bitrate, in bits/minute, was calculated from B, the average number of bits transmitted per block [40,41], using the equations below.

$$B=\log_{2}N+P\log_{2}P+\left(1-P\right)\log_{2}\frac{1-P}{N-1}$$

$$\mathrm{ITR}=\frac{B}{T}$$

where *T* is the average duration of a letter block in minutes (0.69 and 0.28 minutes in RSVP and Matrix, respectively), *N* is the number of possible targets (25 in both modes) and *P* is the probability of accurate letter detection.

Statistical comparisons between conditions of interest were performed using paired t-tests that accounted for potentially unequal variances. The t-value and p-value calculated for each comparison are reported inline with the results below.

## Results

### Behaviour

We compared the accuracy with which participants were able to correctly count occurrences of target letters amongst non-targets in the RSVP and Matrix modes. More specifically, for each letter block, we calculated the absolute difference between the number of times the target letter was presented/flashed and the number of times it was reported as seen. These differences were then averaged separately by subject and mode. Participants saw an average of 86.02% (s.d. = 6.76) and 88.58% (s.d. = 10.57) of targets in RSVP and Matrix modes, respectively. This difference was not significant in a paired t-test (t(1,10) = 0.66, p = 0.52), i.e. participants saw/missed roughly the same proportion of targets in both modes. Hence we concluded that there was no systematic difference in behavioural performance between RSVP and Matrix modes across the participant group.

### Event related potentials

**Figure 2:**
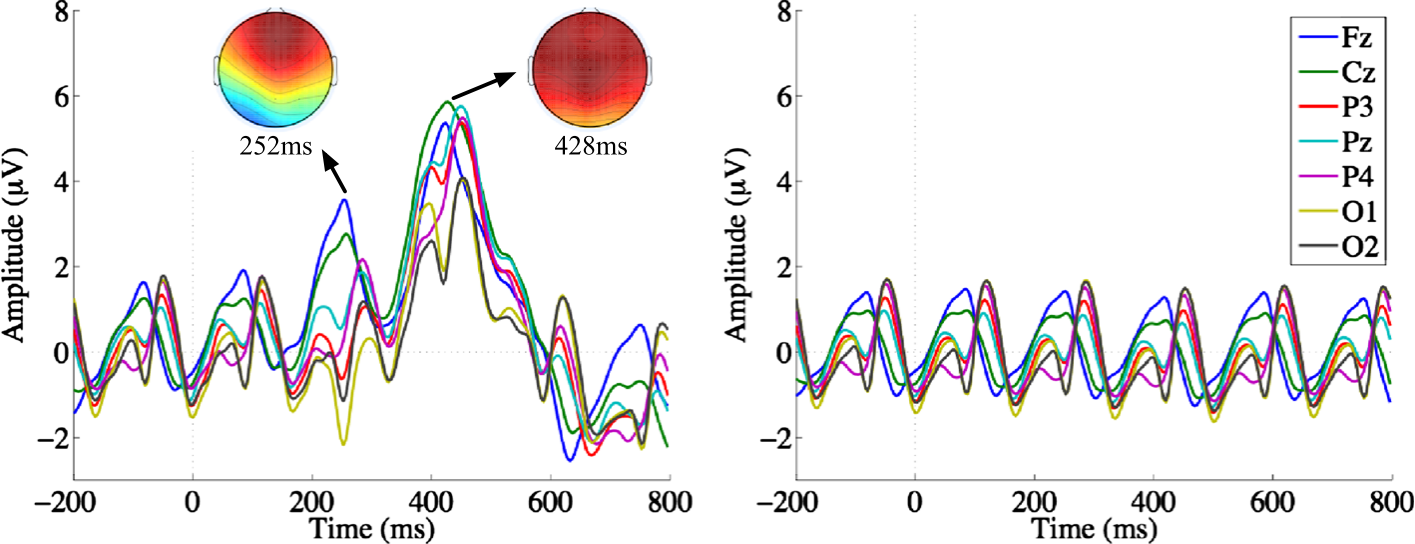
**ERPs evoked by targets (left) and non-targets (right) in RSVP mode.** Targets evoke an early frontal response at 252 ms, followed by a relatively large, distinct parietal P300 b, peaking at 428 ms.

**Figure 3:**
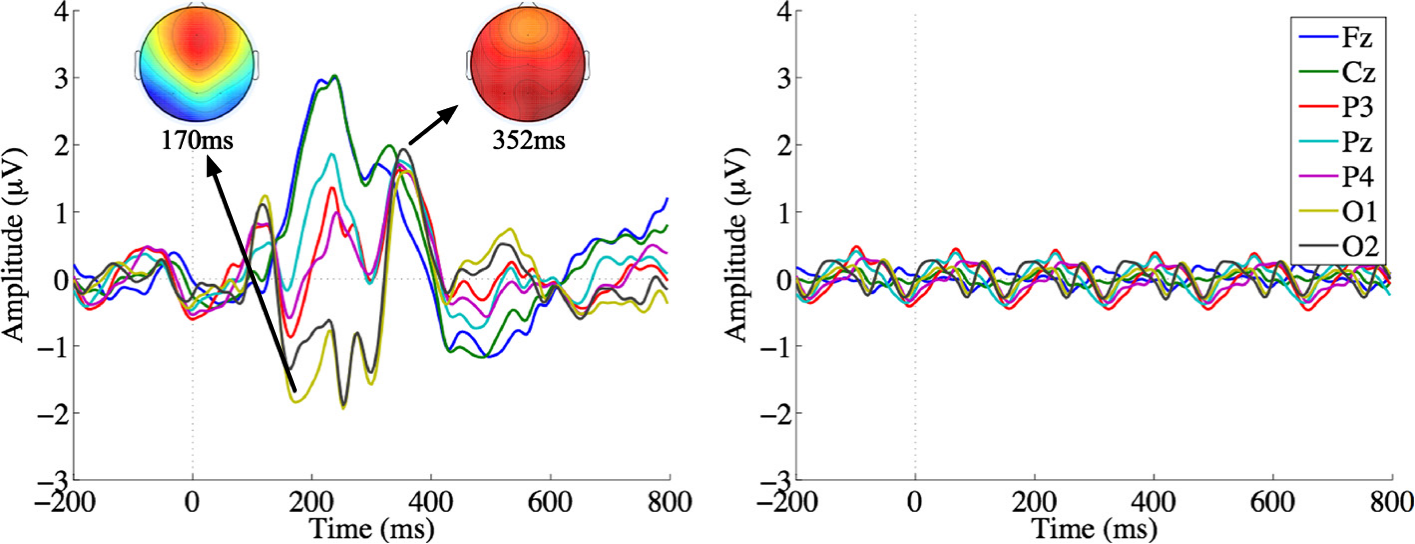
**ERPs evoked by targets (left) and non-targets (right) in Matrix mode.** Targets generate an early Visual Evoked Potential (VEP) with a negative going peak at 170 ms, followed later by a relatively early and small P300 b peaking at 352 ms. These ERPs were generated by ‘pair-averaging’ epochs, one for the row flash, and one for the column flash.

The ERP grand averages at each scalp electrode for targets and non-targets in RSVP and Matrix modes are shown in Figures 2 and 3, respectively. In RSVP mode, targets evoke an early frontal response around 250 ms, followed by a relatively large, distinct parietal P300 b, peaking at 428 ms. In Matrix mode, targets evoked a rather different ERP pattern, similar to those found by [42]: early Visual Evoked Potentials (VEP) with a negative going peak at around 170 ms, followed later by a relatively earlier and smaller P300 b peaking at 352 ms. Note that this Matrix mode ERP was generated by ‘pair-averaging’ epochs, one for the row flash, and one for the column flash (see the Methods section for details).

The observed differences in the ERPs evoked by targets in RSVP and Matrix can be ascribed to key differences in the presentation modes. Users monitored the RSVP stream for a briefly presented target letter. Stimuli in the centrally presented RSVP sequence set up a strong steady-state response (Figure 2, right), which was temporarily interrupted by the relatively larger P300 b evoked by targets (Figure 2, left). On the other hand, participants shifted their gaze to targets that were always visible in the Matrix spellerto detect a change only in luminosity, explaining the pronounced early VEP [42] peaking around 200 ms (Figure 3, left). Further, the P300 b obtained was smaller in Matrix, possibly because target events were more frequent in Matrix (2 flashes out of every 10) than in RSVP (1 presentation out of every 25). However, it should be noted that the effect of stimulus frequency on RSVP P300 b ERPs is yet to be fully characterised in the literature. Of course, there was a cost attached to the more novel targets and larger and hence more discriminable P300 b in RSVP mode: a single repetition took 2.5 times longer, adversely affecting the maximum rate at which letters could be spelt. Next, we investigate how these countervailing influences affect EEG classification and spelling rates.

### Epoch-level EEG classification

**Figure 4:**
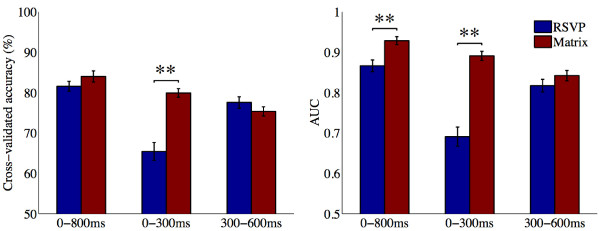
**Epoch classification accuracy (left) and AUC scores (right) in RSVP and Matrix modes.** Figure shows mean and standard error of 10-fold cross-validated epoch classification accuracies and AUC scores. These were calculated by including features within either 0-800 ms, 0-300 ms, or 300-600 ms of each epoch.

**Figure 5:**
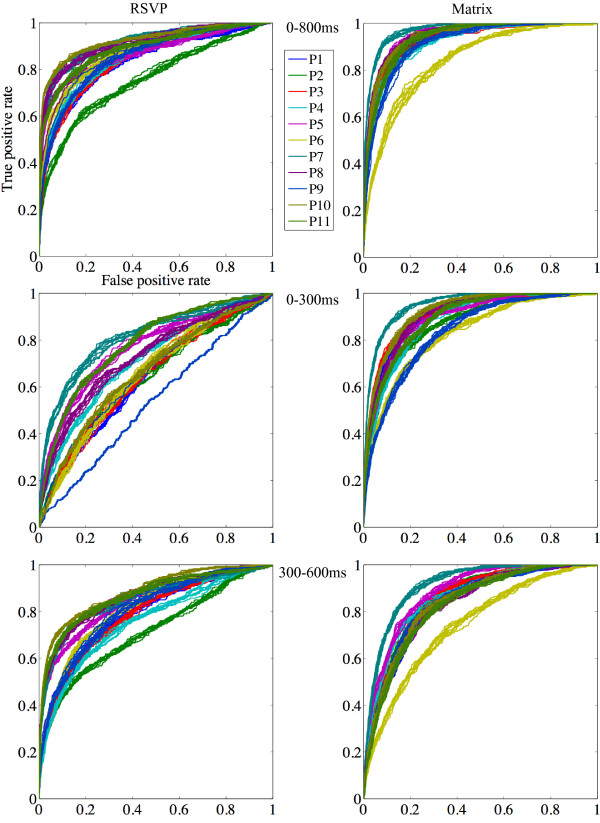
**ROC curves in RSVP (left) and Matrix (right) modes.** Figure depicts subject-wise Receiver Operating Characteristic (ROC) curves for each participant and each cross-validation run, in RSVP and Matrix modes. Curves are coloured by participant, and were calculated by including features within either 0-800 ms, 0-300 ms, or 300-600ms of each epoch.

The mean and standard error (across subjects) of the 10-fold cross-validated classification accuracy of individual epochs in RSVP and Matrix modes are shown in Figure 4. Also plotted alongside are the corresponding Areas Under the ROC Curves (AUC). The individual values for each participant are listed in Tables 1 and 2. The corresponding ROC curves are shown in Figure 5 (top). Note that classification in Matrix mode was performed on ‘pair-averaged’ epochs, which ensured that equal numbers of epochswere included for training and testing in both spelling modes (see the Methods section for details).

**Table 1:** Individual classification accuracies

**Participant**	**RSVP**			**Matrix**		
	**0-800**	**0-300**	**300-600**	**0-800**	**0-300**	**300-600**
1	75.9	55.9	74.0	84.4	81.7	74.7
2	76.9	62.7	73.8	83.3	78.2	76.3
3	75.9	60.3	73.4	85.2	81.9	73.2
4	81.7	69.7	73.2	83.4	77.9	78.9
5	84.0	71.6	78.3	87.5	79.9	79.0
6	80.0	60.0	76.8	72.2	76.8	67.4
7	86.0	75.2	83.4	89.7	87.5	81.5
8	85.3	70.8	82.9	87.2	80.0	73.2
9	80.4	53.8	71.5	81.5	73.8	75.1
10	86.9	65.4	83.8	85.5	82.2	75.7
11	84.3	74.2	82.1	84.3	79.3	73.9

Table lists 10-fold cross-validated epoch classification accuracies for each participant in RSVP and Matrix modes, calculated by including features within either 0-800 ms, 0-300 ms or 300-600 ms of each epoch.

**Table 2:** Individual AUCs

**Participant**	**RSVP**			**Matrix**		
	**0-800**	**0-300**	**300-600**	**0-800**	**0-300**	**300-600**
1	0.83	0.64	0.79	0.93	0.91	0.83
2	0.76	0.64	0.71	0.93	0.87	0.85
3	0.84	0.65	0.79	0.93	0.91	0.85
4	0.85	0.71	0.76	0.92	0.88	0.86
5	0.87	0.77	0.84	0.95	0.89	0.88
6	0.87	0.66	0.82	0.84	0.83	0.74
7	0.92	0.81	0.85	0.97	0.95	0.91
8	0.91	0.73	0.87	0.95	0.91	0.83
9	0.86	0.55	0.81	0.92	0.84	0.84
10	0.93	0.66	0.89	0.94	0.92	0.84
11	0.89	0.79	0.87	0.94	0.89	0.83

Table lists areas under the ROC curve averaged across 10 cross-validation runs for each participant in RSVP and matrix modes, calculated by including features within either 0-800 ms, 0-300 ms or 300-600 ms of each epoch.

The first key finding was that a comparison of classification accuracy when considering all features within the 0-800 ms time window revealed no significant difference between RSVP and Matrix modes (t(1,10) = 1.69, p = 0.12): mean cross-validated accuracies were 81.57% (s.d. = 4.07) and 84.01% (s.d. = 4.54) in RSVP and Matrix, respectively (Figure 4, left). However, AUC scores were significantly higher in Matrix mode (mean = 0.93; s.d. = 0.03) than RSVP (mean = 0.87; s.d. = 0.05): t(1,10) = 3.99, p = 0.003 (Figure 4, right). This difference highlighted the improved discriminability of signal to noise in Matrix epochs, due in part to pair-averaging of epochs in this mode.

In order to measure the differential extents to which early and late ERP components, in particular VEPs and the P300 b, affected classification, we repeated the above analysis, only considering features within either the 0-300ms or the 300-600 ms time windows. We first focus on the results within the 0-300 ms VEP window. As shown in Figure 4 (left), classification accuracies reduced in both modes. But RSVP mode accuracies (mean = 65.42%, s.d. = 7.40) were now significantly lower than Matrix (mean = 79.92%, s.d. = 3.53): t(1,10) = 5.86, p< 0.001. Further, this reduction in accuracy was significantly greater in RSVP than in Matrix: t(1,10) = 6.03, p < 0.001. A similar pattern was observed in the AUC scores with features within 0-300 ms (Figure 4, right; also see ROC curves in Figure 5, middle): mean AUC went down to 0.69 (s.d. = 0.08) in RSVP, but only to 0.89 (s.d. = 0.04) in Matrix, resulting in a large significant difference: t(1,10) = 7.66, p < 0.001. As with the classification accuracy, this decrease in AUC scores was significantly larger in RSVP than Matrix: t(1,10) = 6.3, p < 0.001. This pattern of results is convergent with previous reports of the major influence of gaze-dependent early VEPs in EEG classifiability when using the Matrix P300 BCI [42]. As our findings show, the EEG responses in the RSVP speller carried almost no statistically discriminable information within the 0–300 VEP time window.

Inclusion of features only within the later 300-600 ms P300 b window produced a contrasting pattern of results. As evident in Figure 4 (left), there was no significant difference between the classification accuracies between RSVP (mean = 77.56%, s.d. = 4.74) and Matrix (mean = 75.36%, s.d. = 3.74) in this time window (t(1,10) = 1.29, p = 0.23). In contrast to the 0-300 ms window however, the reduction in accuracy was now significantly greater in Matrix rather than RSVP: t(1,10) = 3.13, p = 0.01. Similarly, AUC scores also reduced in both modes (Figure 4, right; also see ROC curves in Figure 5, bottom), to 0.82 (s.d. = 0.05) in RSVP and 0.84 (s.d. = 0.04) in Matrix. But again, this reduction was significantly higher in Matrix mode than RSVP: t(1,10) = 4.06, p = 0.002. As a result of this differential reduction, the AUC scores were no longer significantly higher in Matrix mode. In other words, during the P300 b time window, the discriminability of signal vs. noise in the two modes were not statistically different. These results complement the pattern observed with the 0-300 ms VEP window: they show that in contrast to Matrix, the RSVP speller is less influenced by bottom-up or exogenously triggered visual ERP components (generated by flashing stimuli in Matrix mode). Rather, it predominantly derives EEG discriminability from the P300 b. We confirmed this directly by measuring the statistically significant effect of the interaction between time window (0-300 ms or 300-600 ms) and spelling mode (RSVP or Matrix) on both classification accuracy (t(1,10) = 8.62, p < 0.001) and AUC scores ((t(1,10) = 7.37, p < 0.001).

### Letter-level EEG classification

**Figure 6:**
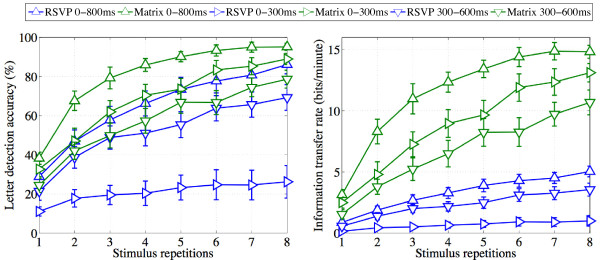
**Letter detection accuracy (left) and ITR (right) in RSVP and Matrix modes.** Figure shows mean and standard error of 50:50 train-test letter detection accuracies and ITRs as a function of the number of stimulus repetitions. These were calculated by including features within either 0-800 ms, 0-300 ms, or 300-600 ms of each epoch.

In order to estimate the extent to which offline performance evaluated in the previous section might generalise to an online BCI setting, we used a 50:50 train-test procedure to calculate the average number of letters correctly identified in each presentation mode. Figure 6 depicts the letter detection accuracy and ITR in RSVP and Matrix modes as a function of the number of stimulus repetitions included for detection. The individual values for each participant are listed in Tables 3 and 4. As described in the Methods section, letter detection accuracy was calculated using a 50:50 train-test procedure.

**Table 3:** Individual letter detection accuracies

**Participant**	**RSVP**			**Matrix**		
	**0-800**	**0-300**	**300-600**	**0-800**	**0-300**	**300-600**
1	87.5	6.2	68.8	93.8	93.8	93.8
2	46.7	0.0	26.7	93.3	86.7	86.7
3	93.3	6.7	60.0	100.0	91.7	58.3
4	86.7	20.0	60.0	100.0	73.3	80.0
5	93.3	60.0	80.0	93.3	93.3	80.0
6	86.7	0.0	73.3	80.0	80.0	46.7
7	100.0	80.0	80.0	93.3	93.3	86.7
8	100.0	40.0	93.3	100.0	100.0	100.0
9	73.3	6.7	40.0	100.0	73.3	73.3
10	100.0	13.3	100.0	93.3	93.3	80.0
11	80.0	53.3	80.0	100.0	100.0	80.0

Table lists 50:50 train-test letter detection accuracies for each participant in RSVP and Matrix modes, calculated by including features within either 0-800 ms, 0-300 ms or 300-600 ms of each epoch.

**Table 4:** Individual ITRs

**Participant**	**RSVP**			**Matrix**		
	**0-800**	**0-300**	**300-600**	**0-800**	**0-300**	**300-600**
1	5.0	0.0	3.3	14.2	14.2	14.2
2	1.7	0.0	0.6	14.1	12.3	12.3
3	5.7	0.0	2.6	16.3	13.6	6.2
4	4.9	0.4	2.6	16.3	9.2	10.7
5	5.7	2.6	4.3	14.1	14.1	10.7
6	4.9	0.0	3.7	10.7	10.7	4.3
7	6.5	4.3	4.3	14.1	14.1	12.3
8	6.5	1.3	5.7	16.3	16.3	16.3
9	3.7	0.0	1.3	16.3	9.2	9.2
10	6.5	0.1	6.5	14.1	14.1	10.7
11	4.3	2.1	4.3	16.3	16.3	10.7

Table lists 50:50 train-test information transfer rates for each participant in RSVP and Matrix modes, calculated by including features within either 0-800ms, 0-300ms or 300-600ms of each epoch.

We first compared letter detection accuracies when considering all features within the 0-800 ms window. As can be seen in Figure 6 (left), though accuracy in both modes increased as more repetitions were included, Matrix outperformed RSVP mode when fewer epochs were included. In addition, this increase in accuracy tended to asymptote in both modes, reaching 86.14% in RSVP (s.d. = 15.63) and 95.19% in Matrix (s.d. = 6.02) when 8 repetitions were used for testing (rightmost points of plots in Figure 6). At this point, there was no longer any significant difference in accuracy between the modes: t(1,10) = 1.8, p = 0.1. Extrapolating this finding to an online BCI setting, the SWLDA classifier would have been able to detect the letter the participant was trying to spell equally well in the two modes.

The calculation of letter detection accuracy did not, however, take into account the lower target presentation frequency in RSVP mode. This was captured by the Information Transfer Rate (ITR) or bitrate, which highlighted the large difference in effective communication speed between the two modes (Figure 6, right). As would be expected, the ITRs increased as more repetitions were included, levelling off at significantly different values: 5.03 bits/minute (s.d. = 1.45) and 14.83 bits/minute (s.d. = 1.76) in RSVP and Matrix respectively (t(1,10) = 14.28, p < 0.001). This finding can be attributed to the key difference between the two modes: the gaze/space dependence entailed by the Matrix speller means that the presentation of a single repetition is much shorter (1.66 s). In comparison, the RSVP speller sacrifices space, and requires 4.15 s to present a single repetition (see sections RSVP mode and Matrix mode in Methods for details).

To further investigate these trade-offs involved in space (in) dependence, we evaluated the role of ERP time windows in driving letter detection accuracy and ITR. Figure 6 plots these measures when performing a 50:50 train-test procedure only on features within 0-300 ms or 300-600 ms. With the shift to the 0-300 ms window, detection accuracy dropped dramatically in RSVP mode (Figure 6, left).There was no asymptotic increase with additional stimulus repetitions, resulting in mean detection accuracy of only 26.02% (s.d. = 27.75) after 8 repetitions. Accuracy in Matrix mode, however, still showed an asymptotic increase, reaching a significantly higher value of 88.98% (s.d. = 9.51) after 8 repetitions (t(1,10) = 7.12, p < 0.001). The relative reductions in detection accuracy were also significantly different: t(1,10) = 6.58, p < 0.001. As would be expected, mean ITR within the 0-300 ms window (Figure 6, right) in RSVP also remained low at 0.99 bits/minute (s.d. = 1.44), while it reached 13.12 bits/minute (s.d. = 2.51) in Matrix, resulting in a large significant difference (t(1,10) = 13.91, p < 0.001). The relative reduction in ITR was also significantly different between the two modes: t(1,10) = 2.39, p = 0.03. Taken together, these findings reiterate the point that ERPs in RSVP mode carried relatively little statistically discriminable information within the 0–300 VEP time window. Hence the RSVP speller relys almost entirely on the P300 b to drive performance.

A contrasting pattern was found on inclusion of features within the 300–600 P300 b time window. As can be seen in Figure 6, letter detection accuracies and ITRs were adversely affected in both modes, but the Matrix mode was clearly more affected by the change from 0-800 ms to 300-600 ms. After 8 repetitions, mean detection accuracies were 69.28% (s.d. = 21.75) in RSVP and 78.67% (s.d. = 15.11) in Matrix. These means were not statistically different (t(1,10) = 1.18, p = 0.26), nor were the relative reductions in their values when compared to the 0-800 ms window. ITRs after 8 repetitions were also reduced with the 300-600 ms time window, to 3.56 bits/minute (s.d. = 1.74) in RSVP and a significantly higher value of 10.69 bits/minute (s.d. = 3.36) in Matrix (t(1,10) = 6.25, p < 0.001). Importantly, in contrast to the 0-300 ms window, this reduction in ITR was significantly larger in Matrix than RSVP: t(1,10) = 2.66, p = 0.02. Hence, as with the cross validation analysis, we observed a significant interaction between spelling mode (RSVP vs. Matrix) and analysis window (0-300 ms vs. 300-600 ms), on both letter detection accuracy (t(1,10) = 6.15, p < 0.001) and ITR (t(1,10) = 4.69, p = 0.002). Again, this highlighted the dependence of Matrix mode performance on early VEPs and RSVP mode performance on late P300 b ERPs. The overall pattern of results with 50:50 train-test analysis are qualitatively similar to those obtained with cross validation, suggesting that this pattern would be likely to carry over to online performance.

## Discussion

We have motivated interest in completely space-independent BCIs, particularly emphasising that deficits associated with overt or covert attentional shifts may make anything other than a foveally bound presentation unfeasible. RSVP BCI designs described relatively recently [31-34] have demonstrated its viability for developing space-independent BCI applications. The key design difference in RSVP that enables space independence is that all selection alternatives are presented at fixation and selections are detected as perceptual breakthroughs indexed by the P300 ERP. From a cognitive perspective, a brief sketch of the processes involved in detecting a target in RSVP is as follows. Firstly, a template of the stimulus being consciously searched for (e.g. the letter ‘K’ in a BCI) is instantiated into and then held in a task set, becoming an effective ‘target’ for that search. The vast majority of non-targets are rejected sub-threshold, i.e. without engaging awareness. However, when a match to the target template is registered, stimulus representations in the brain are enhanced, generating a conscious percept, which is electro physiologically marked by a P300; see [29,43,44] for a neural theory formalising this information processing sketch.

In this work, we have compared RSVP-based spelling to the well-established letter matrix design. We have done so in an offline, within-subject setting, while keeping all other parameters identical for a fair comparison. The principal finding of this comparison is that both designs deliver roughly the same level of accuracy in detecting user selection. In the context of fully space-independent BCIs, we have demonstrated that the RSVP approach provides a significantly higher throughput than an existing method, the overlaid gratings approach described in Allison *et al*. [26]. Specifically, RSVP achieved a bit rate around 5 bits/minute. This throughput is similar to that achieved by the online RSVP speller tested by [33], and improves upon the SSVEP-based space-independent BCI tested by Allison *et al*. [26], which obtained 1 bit/min or less. However, as would be expected, the Matrix speller outperforms RSVP in terms of spelling throughput, due to its exploitation of space to speed up stimulus presentation. As we have shown, the flipside of this is that space-dependent VEPs have significantly greater influence on EEG classification in the Matrix speller. This result informs the consequent trade-offs entailed by RSVP vs. Matrix BCI designs for potential applications with patients, depending on the severity of their impairment in directing gaze or attention.

In a valuable experiment [42], compared the performance of the Matrix speller when participants were allowed to move gaze and attention in space, to when they were required to fixate centrally and covertly attend to target flashes in the letter matrix. They found a severe reduction in EEG classification accuracy when only covert attentional shifts were allowed. In our comparison of RSVP vs. Matrix, we did not require our participants to fixate centrally in the Matrix mode. The main reason for this was because we aimed to estimate the performance costs resulting from space independence, by evaluating BCIs at either end of a potential spectrum. Yet another reason, as pointed out by [42] themselves, is that requiring central fixation in Matrix mode would have significantly increased the cognitive load (because of having to fixate centrally while attending peripherally) relative to RSVP. As in their study, this would have severely reduced performance in Matrix mode and resulted in an unequal comparison.

When considering real-world applications of RSVP spellers, it is important to note that the RSVP speller presented here is a prototype. In particular, many parameters of the design are ripe for optimisation. No mode-specific optimisation was performed here, as the aim was to ensure an equal comparison between RSVP and Matrix, in the sense that all other presentation parameters were kept the same. Amongst those that could be optimised, perhaps most significant is the SOA parameter, fixed here at 166 ms in both modes. This is effectively an arbitrary choice, which has a major impact on the bit-rate obtained. In fact, most theoretical studies have used faster presentation (typically with an SOA of ~100 ms), and still obtained good single target accuracies, often between 80-90%. Indeed, even increasing presentation rate beyond 10 Hz has been reported to result in relatively small decrements in accuracy. For example [29], found a 20% drop in accuracy when SOA fell from 100 ms to 50 ms. So, it may be possible to cut the presentation time considerably with only a relatively small decrement in behavioural accuracy. However, the effect this would have on P300 size and profile remains a question for further empirical study. Stimulus features that make letters more discriminable from each other constitute another form of optimisation for improving RSVP spelling rates. For example [31,33], have shown that enhancing differences between letters by altering their colour and/or shape can influence classification performance.

One alternative to presenting a full alphabet of letters in RSVP would be to present only the 10 row and column numbers from the Matrix speller in RSVP [45]. In order to spell a letter, the user would have to detect occurrences of the numbers identifying the row and column containing the letter they want to spell. Though this alternative would probably require more user training, it would make the duration of a repetition (and hence ITR) in RSVP identical to that in Matrix. An alternative to this idea is the ‘Center Speller’ [19], which employed a two-stage approach for a similar speedup of presentation rate: in the first stage, users selected one of many letter groups presented serially, in a circle around fixation. Once a letter group selection was detected, only letters from that group were then displayed in a circle, and users selected one amongst them to complete the second stage. In addition to such optimisations, significant improvements in spelling can be generated by exploiting potential synergies between classification algorithms coupled with adaptive error correction techniques and predictive language models. For example [34,46], have demonstrated the value of fusing EEG classification with language modelling to predict the word being spelt. The generation and adaptive updating of user-specific language dictionaries are likely to further improve the efficacy of this technique. Further improvements in the usability of BCIs are likely with the incorporation of asynchronous operational capabilities [47].

Other RSVP stimulus presentation issues remain and require empirical clarification. For example, is there a bit rate difference between regular and randomised ordering of stream stimuli? The former, due to its predictability, is probably easier for the user, while the latter, due to its unpredictability, possibly elicits a larger P300. There are also a number of psychophysical findings that potentially impact the RSVP speller, these include the attentional blink [28,29] and repetition blindness [43,48]. These could be used to constrain the structure of RSVP streams, such that, for example, a priori frequently occurring letters do not appear in one another’s blink window and the next instance of an item does not arise within the repetition blindness window of a previous instance. Indeed, many of the issues relating to presentation format arise generally across applications of such ‘subliminal salience search’ mechanisms [49], and their empirical resolution could have broad impact. This mechanism could be applied in lie detection [49], information retrieval, image triage [50] and stimulus rich information presentation [51]. Further understanding of presentation parameters and their influence on EEG responses could benefit all such applications.

Finally, it is worth considering that non-visual forms of BCI designs might also be suitable for some patients unable to direct either overt gaze or covert attention in visual space. Auditory and tactile modalities have been explored as means to replace visual stimulation in such cases (see [35] for a review). Such BCIs usually achieve lower ITRs in comparison to the Matrix speller due to the relatively lower ‘bandwidth’ available for presenting information in auditory/visual modalities. However, locked-in patients have reported difficulties concentrating on stimuli in an auditory instantiation of the Matrix speller [52], suggesting that simpler spelling interfaces might be required to match their attentional capabilities. Nevertheless, non-visual BCIs might still be viable for achieving gaze and space independence, albeit with simpler tasks that reduce cognitive load while sacrificing high bitrates [35].

## Conclusions

The empirical work presented here has provided a comparative assessment of accuracy and efficacy of RSVP and Matrix P300-based BCI spellers. These two spellers are positioned at either ends of a spectrum of BCI designs with varying degrees of space independence. We find that both designs perform equally well in terms of detecting the user’s selection. Our comparison dwells on the trade-offs inherent in the choice between these designs: fully space-independent RSVP designs are less efficient, in terms of spelling rate, than gaze and space-dependent Matrix designs. However, RSVP designs are also less reliant on early space-dependent VEPs to drive classification accuracy, which is a key consideration for users unable to shift gaze or attention in space. With key improvements to the RSVP design, true space-independent BCIs could approach efficiencies on a par with the Matrix speller, making it a viable alternative for such users.

## Competing interests

The authors declare that they have no competing interests.

## Authors’ contributions

SC designed and implemented the study, and wrote the manuscript. ASA helped design and run the study, and contributed to the manuscript. MF helped run the study. AMO supervised the research and contributed to the manuscript. HB supervised the research, helped design the study and helped write the manuscript. All authors read and approved the final manuscript.
